# Spontaneous Clearance of *Mycobacterium ulcerans* in a Case of Buruli Ulcer

**DOI:** 10.1371/journal.pntd.0001290

**Published:** 2011-10-25

**Authors:** Claire L. Gordon, John A. Buntine, John A. Hayman, Caroline J. Lavender, Janet A. Fyfe, Patrick Hosking, Paul D. R. Johnson

**Affiliations:** 1 Department of Infectious Diseases, Austin Health, Melbourne, Australia; 2 Department of Surgery, Box Hill Hospital, Melbourne, Australia; 3 Department of Surgery, Monash University, Melbourne, Australia; 4 Department of Anatomy and Developmental Biology, Monash University, Melbourne, Australia; 5 WHO Collaborating Centre for Mycobacterium ulcerans (Western Pacific Region) and Victorian Infectious Diseases Reference Laboratory, Melbourne, Australia; 6 Department of Pathology, Box Hill Hospital, Melbourne, Australia; University of Tennessee, United States of America

Buruli ulcer (BU) is an infection of skin and soft tissue caused by *Mycobacterium ulcerans*, a toxin-producing environmental mycobacterium. Significant advances in the treatment of BU have been made over the past decade with the introduction of effective antibiotic therapy and there is a greater understanding of the pathogenesis and host immune response. Although it is generally held that early BU lesions may heal spontaneously, to our knowledge, there are no previously published cases that definitively document spontaneous resolution of culture-confirmed BU.

## Presentation of Case

**Figure 1:**
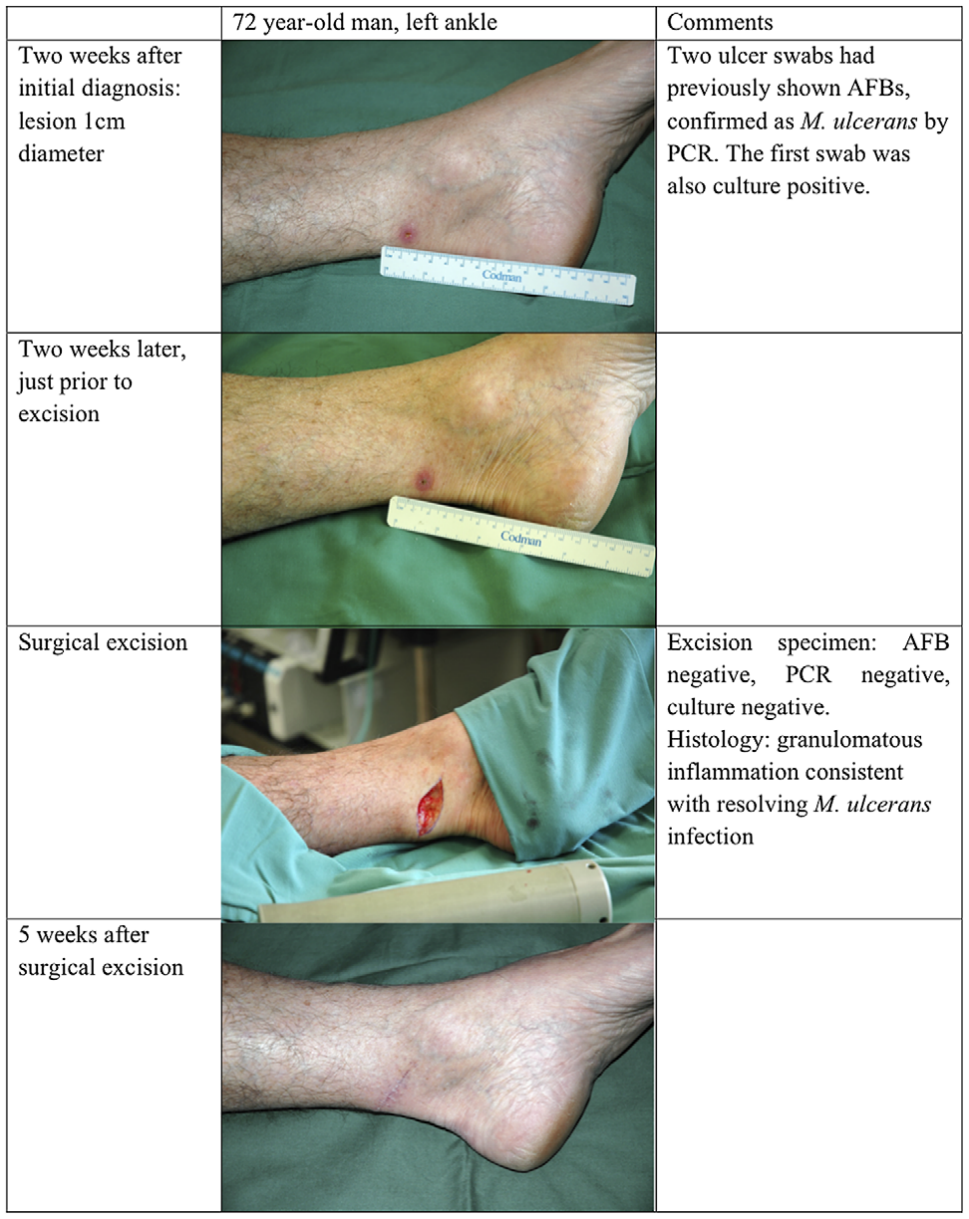
Clinical appearance and laboratory characteristics.

The patient was a 72-year-old man from Melbourne, Australia, who had visited his holiday house in Point Lonsdale, an endemic area of BU on the coast 70 km to the southwest [1], [2], most weekends for the past 28 years. He had a past medical history of coronary artery disease but was otherwise fit and active. In October 2008 he noted a 5-week history of a slowly progressive painless papule with a punctate centre on his left ankle (Figure 1). His most recent visit to Point Lonsdale was 2 weeks before the lesion appeared.

**Figure 2:**
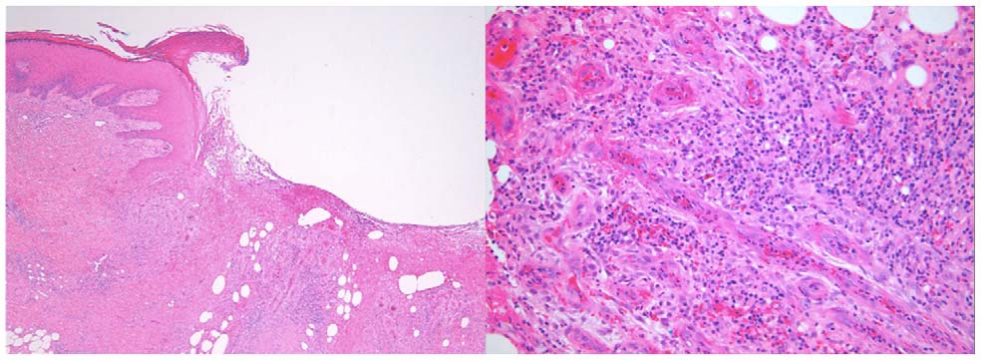
Histopathology of excised lesion. Left panel: Section of excised skin ulcer, showing one ulcer margin with fibrinous exudate in the base of the ulcer. Granulation tissue with a mixed inflammatory cell infiltrate extends into subcutaneous fat (H&E, orig mag ×40). Right panel: Section from the base of the ulcer, showing granulation tissue with a mixed inflammatory cell infiltrate. Acid-fast bacilli were not seen in a Z-N stained section of the same area (H&E, orig mag ×200).

The diagnosis of BU was made unconventionally. The patient's 12-year-old grandson was attending a clinic with his mother for treatment of IS*2404* PCR-confirmed [3] BU on his lower back as reported elsewhere [4]. During the consultation she mentioned that her father also had a progressive lesion on his ankle. She was given a dry specimen swab to take home with instructions on how to sample her father's ankle lesion. Material from the ankle swab tested positive for acid-fast bacilli (AFB) by Ziehl-Neelsen (Z-N) staining and *M. ulcerans* infection was confirmed by PCR and later by culture. A repeat swab taken by the treating clinician (PDRJ) 2 weeks later was again positive for AFB, and PCR again confirmed *M. ulcerans* infection. Culture from this second specimen was ultimately negative, although the culture result was not available at the time management decisions had to be taken. Antibiotics were considered but not prescribed [5], as there was a potential interaction between the patient's cardiac medications and rifampicin. Instead, excision with primary closure was performed without the use of antibiotics or other treatment modalities (Figure 1). Sections of the excised tissue showed a 3-mm area of skin ulceration (Figure 2, left panel) with mixed inflammatory cell infiltration and granulation tissue extending into subcutaneous fat (Figure 2, right panel). AFB were not detected, but the appearances were considered consistent with *M. ulcerans* infection in a healing or resolving phase of the infection [6]. The excised tissue specimen was divided into five sub-sections and all portions screened by both PCR and culture according to standard operating procedures. Generally, this means that the entire sub-section of tissue is cut into smaller pieces and homogenised in a bottle containing glass beads and 2 ml of phosphate buffered saline. If the sub-section is too large to fit into the bead bottle, smaller pieces of tissue are taken from throughout the specimen to maximise the likelihood of sampling organisms. One ml of tissue homogenate is then processed for PCR [3] and 1 ml used for culture in BACTEC 12B bottles and on Brown and Buckle medium [7] incubated at 31°C for up to 12 weeks. *M. ulcerans* was no longer detected by either method. The patient gave informed consent for publication.

## Case Discussion

Buruli ulcer has an alarming potential for progressive tissue destruction and has the potential to leave patients permanently disabled due to widespread necrosis of subcutaneous fat, extensive fibrous scar tissue formation, and contractures [6], [8], [9]. However, even when no effective therapy is available, progression may cease [10], [11]. The human immune system is therefore able to contain *M. ulcerans*, albeit after some years and considerable tissue destruction. The details of how this final victory is won are of great importance to researchers working on BU vaccines.

Our patient first noted a lesion 5 weeks before the first diagnostic specimen was obtained. AFB were seen, IS*2404* PCR was positive, and *M. ulcerans* was isolated by culture. The diagnosis was reconfirmed with a second swab. The lesion was excised a month later but no AFB were detectable by then, and both PCR and culture were negative. Of note, the patient's grandson who visited his grandfather's house at Point Lonsdale contemporaneously developed a large progressive BU over his lower back [4].

*M. ulcerans*, like *M. tuberculosis* and *M. leprae*, does not cause clinical disease in all exposed individuals. Gooding et al. [12] showed that the prevalence of antibodies to *M. ulcerans* in exposed household controls in Queensland was similar to that in patients with proven BU using a whole cell antigen preparation. Similar findings with more specific antigens have confirmed this observation in West Africa [13], [14]. However, antibodies to *M. ulcerans* are not *M. ulcerans* specific and are encountered in persons from endemic and non-endemic regions, which makes it difficult to evaluate evidence for spontaneous remission based on the presence of antibodies. As with most infectious diseases, variation in host immune response genes is likely to influence susceptibility. In a study performed in Ghana, the SLC11A1 (NRAMP1) D543N polymorphism, which confers susceptibility to tuberculosis and leprosy, has been linked to increased susceptibility to BU, with an estimated 13% population attributable risk [15].

However, it also likely that inoculum size influences outcome. For example, our patient may have received a lower initial inoculum than his grandson, although the natural inoculum size has not been definitively established. Fenner noted that the protective effect of BCG against *M. ulcerans* in a mouse model could be overcome by increasing the inoculum [16]. Unlike the grandson, it is also possible that our patient had a prior exposure to *M. ulcerans* that may have immunised him and enhanced his ability to control his infection, as he reported very frequent visits to Point Lonsdale where transmission of *M. ulcerans* has been common since at least 2002 [1], [2].

This report of spontaneous clearance of *M. ulcerans* from a small but clinically apparent BU confirms previous anecdotal and some systematic field observations in patients likely to have BU, but from whom definitive laboratory confirmation was not available [17]. For example, Revill et al. reported that 29% of patients with small nodular lesions diagnosed clinically healed spontaneously while receiving placebo during a randomised study of clofazamine therapy [18].

The key virulence factor of *M. ulcerans*, mycolactone, has potent cytotoxic and immunosuppressive properties that act both locally and systemically. Initially, the histology of BU lesions shows bland necrosis with a remarkable absence of an acute inflammatory response (reviewed in Schütte et al. [19]). However, natural halting of progressive infection, or treatment with antibiotics, appears to be associated with the development of a vigorous Th-1 response, the development of delayed type hypersensitivity to mycobacterial antigens, and intense granulomatous inflammation on histology (reviewed by Demangel et al. [20] and Schütte et al. [19]). At present, no satisfactory model exists to explain how the human host is able to sterilize active *M. ulcerans* infections that are likely to be producing increasing local concentrations of mycolactone, particularly as mycolactone itself does not appear to stimulate the production of neutralising antibodies [19]. The mechanism by which this occurs will be of great interest, as a vaccine able to induce this sterilizing response should be highly effective for both primary prevention and as an adjuvant to therapy.

##

**Key Learning Points**

- Some people with confirmed Buruli ulcer are able to spontaneously eradicate *M. ulcerans* before they develop destructive lesions.
- This suggests a major role for adaptive immunity in protection against *M. ulcerans* infection.
- When new treatments for *M. ulcerans* are evaluated the possibility of spontaneous resolution needs to be considered.
